# Photoactive and conductive biohybrid films by polymerization of pyrrole through voids in photosystem I multilayer films†‡

**DOI:** 10.1039/d3na00354j

**Published:** 2023-08-26

**Authors:** Joshua M. Passantino, Blake A. Christiansen, Marc A. Nabhan, Zane J. Parkerson, Tyler D. Oddo, David E. Cliffel, G. Kane Jennings

**Affiliations:** a Department of Chemical and Biomolecular Engineering, Vanderbilt University Nashville TN 37235-1604 USA kane.g.jennings@vanderbilt.edu; b Department of Chemistry, Vanderbilt University Nashville TN 37235-1822 USA

## Abstract

The combination of conducting polymers with electro- and photoactive proteins into thin films holds promise for advanced energy conversion materials and devices. The emerging field of protein electronics requires conductive soft materials in a composite with electrically insulating proteins. The electropolymerization of pyrrole through voids in a drop-casted photosystem I (PSI) multilayer film enables the straightforward fabrication of photoactive and conductive biohybrid films. The rate of polypyrrole (PPy) growth is reduced by the presence of the PSI film but is insensitive to its thickness, suggesting that rapid diffusion of pyrrole through the voids within the PSI film enables initiation at vacant areas on the gold surface. The base thickness of the composite tends to increase with time, as PPy chains propagate through and beyond the PSI film, coalescing to exhibit a tubule-like morphology as observed by scanning electron microscopy. Increasing amounts of PPy greatly increase the capacitance of the composite films in a manner almost identical to that of pure PPy films grown from unmodified gold, consistent with a high polymer/aqueous interfacial area and a conductive composite film. While PPy is not photoactive here, all composite films, including those with large amounts of PPy, exhibit photocurrents when irradiated by white light in the presence of redox mediator species. Optimization of the Py electropolymerization time is necessary, as increasing amounts of PPy lead to decreased photocurrent density due to a combination of light absorbance by the polymer and reduced accessibility of redox species to active PSI sites.

## Abstract 2

Electropolymerization of pyrrole (Py) through drop-casted films of photosystem I (PSI) protein leads to photoactive and electrically conductive composite films.

## Graphical abstract

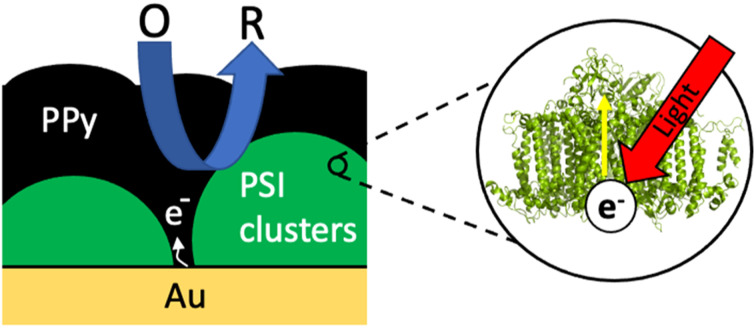

## Introduction

Photosystem I (PSI) is an integral *trans*-membrane protein complex that is instrumental in photosynthesis. *In vivo*, the primary function of PSI is to accept low-energy electrons from plastocyanin, excite the electrons to a higher energy state with photons collected by surrounding chlorophylls, and donate the excited electrons to ferredoxin to produce NADH.^1^ Because PSI is abundant in nature and approaches 100% quantum efficiency from the light it harvests,^2^ many investigators are exploring this protein as a prominent candidate bionanomaterial for biohybrid solar energy conversion. The PSI protein complex is a powerful redox-active biological material that can be incorporated with a host of other materials, including porous electrodes,^3,4^ semiconductors,^5,6^ redox polymers,^7,8^ conducting polymers,^9,10^ metal–organic frameworks,^11^ nanoparticles,^12,13^ carbon nanotubes,^14,15^ and graphene,^16–18^ to name several.

The photoresponsive oxidation and reduction capabilities of PSI, as well as its rapid charge separation, are leading factors that attest to its suitability for photoelectrochemical applications,^19^ similar to those shown by other nanomaterials in artificial photosynthesis.^20–22^ On the stromal side of the protein, the iron–sulfur cluster known as the F_B_ site attains one of the most negative reduction potentials (−590 mV *vs.* SHE) in nature.^23^ In the lumenal pocket, the P_700_ chlorophyll pair has been shown to have a potential near 450 mV *vs.* SHE, enabling P_700_ to oxidize a wide range of electron donors. PSI drives a potential difference of over 1 V across the thylakoid membrane with >99% energy conversion efficiency of the light absorbed by the protein.^23^

Since PSI has an insulating periphery with redox active sites near its lumenal and stromal surfaces, the direct connection to those active sites can lead to improved solid-state biohybrid systems. Several researchers have sought to embed the protein within a conductive polymer matrix in attempts to provide more direct connections to transfer electrons to/from the active sites of PSI. Examples of successful strategies include immobilization of PSI in an Os-rich redox polymer hydrogel,^7^ a Zn-rich metal organic framework,^11,24^ and a copolymer containing intercalated and conjugated oligoelectrolytes.^25^ Other approaches have included layer-by-layer deposition of PSI with poly(benzyl viologen)^26^ or poly(3,4-ethylenedioxythiophene):polystyrene sulfonate (PEDOT:PSS);^27^ vapor-phase polymerization of EDOT within a PSI multilayered film;^10^ deposition of multilayers containing PSI along with polytriarylamine,^28^ PEDOT:PSS,^29^ or polyxylylviologen;^30^ spin coating of films from mixtures of PSI and PEDOT:PSS;^31^ and entrapment of PSI proteins from solution within an electrochemically grown polyaniline film.^9,32^ In most of these approaches, combining PSI with the redox or conducting polymers and other components increases the photoconversion performance of the film or device beyond those of PSI or the polymer alone. These enhancements have been attributed to protein-polymer interactions that have facilitated electron transfer to/from the protein.^27,31,32^ However, much remains to be discovered about the types of PSI-polymer interfaces that yield these synergistic results. The development of new methods for fabricating composite PSI-polymer films can lead to unique structures and architectures to investigate this important protein-polymer interface.

In our first attempt to pair PSI with polypyrrole (PPy), we have recently shown that PSI can photooxidize pyrrole (Py) in solution to generate PSI-PPy bulk composites that exhibit light-enhanced conductivity.^33^ The ability of PSI to photooxidize Py suggests that the energy levels between the protein and polymer are well matched. However, the insolubility of the PPy-rich composite yielded discontinuous composite films.

**Fig. 1:**
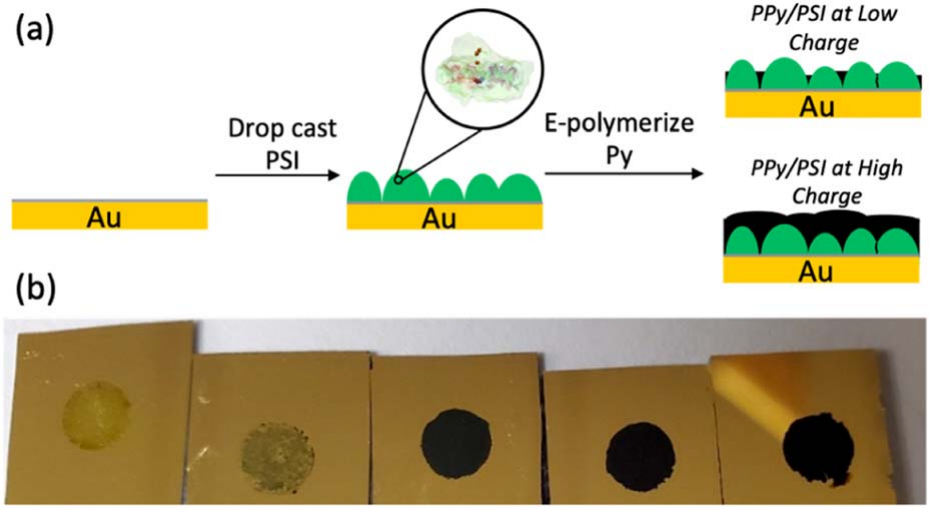
(a) Steps in preparing PSI-PPy composite films by electropolymerization of Py from gold through voids in a drop-cast PSI film. (b) Visual appearance of PSI multilayer films after polymerization of Py. From left to right, the charge density passed during polymerization is 0, 14, 70, 140, and 210 mC cm^−2^.

In this work, we report a more straightforward approach to combine PSI and PPy into dense, continuous films in which the insolubility of PPy is an advantage to stabilize the composite. In this approach, we can also embed high concentrations of PSI into a conducting polymer matrix to fabricate conductive and photoactive films. First, a thick multilayer of PSI is drop cast onto a gold surface, and Py is electrochemically polymerized into PPy through voids in the protein film (Fig. 1a). This method enables high concentrations of protein within the film and yields conductive biohybrid films in which PSI clusters are separated from PPy domains. The effects of the PSI film on the electropolymerization kinetics, as well as the effects of the amount of PPy deposited on the pseudocapacitance and photoelectrochemical performance of the biohybrid composite films are examined. The results of this research, compared with those of other published approaches for fabricating PSI-polymer composite films, provide insight into both the importance of protein-polymer interactions to maximize photoelectrochemical performance and the aptness of the film fabrication method to facilitate those.

## Results and discussion

Composite PPy-PSI films were fabricated as shown in Fig. 1a. Multilayer films of PSI were drop cast onto gold substrates into a known geometric area framed by an electrochemical mask. These substrates were then exposed to Py monomer solution and were biased at a set potential until a specified amount of PPy was grown, based on the charge passed through the electrode. Representative visual images of the resulting films are shown in Fig. 1b. Visually, the pure multilayer of PSI is a non-uniform, translucent, green film that is characteristic of the drop casting approach.^31^ After 14 mC cm^−2^ of PPy is deposited, the film remains visually non-uniform but becomes less transparent and a darker shade of green, characteristic of limited growth of PPy. At 70 mC cm^−2^ and beyond, the entire surface appears covered by PPy and is completely opaque.

### Film composition

**Fig. 2:**
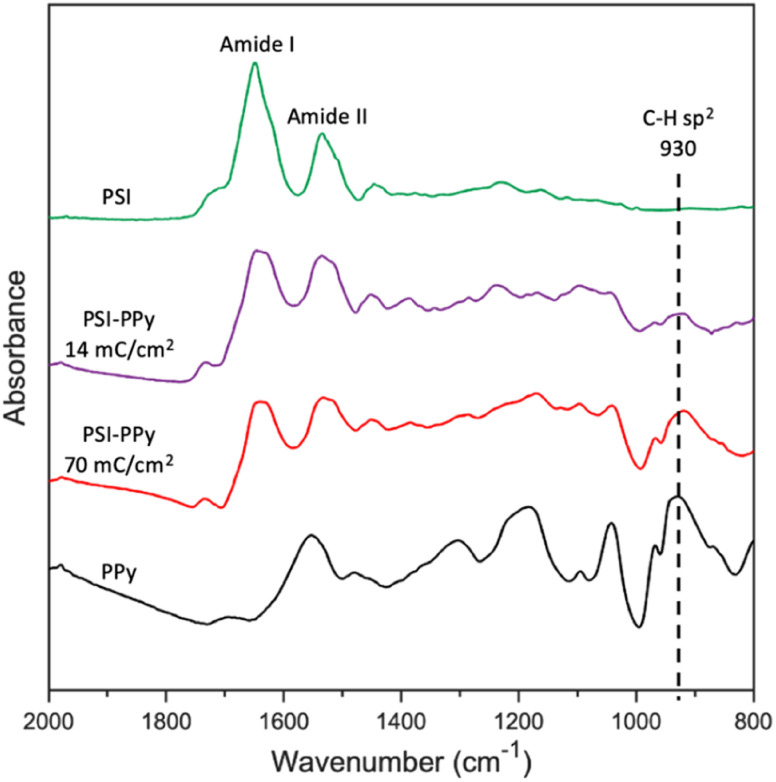
ATR FTIR spectra of PSI, PPy, and two PSI-PPy composite films with different amounts of deposited polymer. The substrate electrode is gold. The spectra have been offset vertically for clarity.

ATR-FTIR analysis was used to confirm the presence of both PSI and PPy in electropolymerized films. Fig. 2 shows the characteristic spectra for films of pure electropolymerized PPy with significant peaks at 1555 (N–H bending) and 930 (C–H sp^2^ bending) cm^−1^, films of pure PSI with peaks at 1656 (Amide I) and 1531 (Amide II), and two composite films in which Py was electropolymerized to charges of 14 and 70 mC cm^−2^ at a potential of 0.65 V (*vs.* Ag/AgCl) through the voids in a film of PSI. Most notably, the spectra for the composite PSI-PPy films show a peak at 930 cm^−1^ due to C–H sp^2^ bending of PPy while retaining the Amide I and II peaks of PSI. With the addition of more Ppy, the peaks attributed to Amide I and II in the spectrum of PSI become broader, and the valley between them shallower, due to growth of the aromatic N–H peak as in the PPy spectra. The ratio of the height of the Amide I peak to that evolving at 1540 cm^−1^ decreases from 1.71 to 0.98 as the amount of charge passed is increased to 70 mC cm^−2^, consistent with the presence of more PPy. Importantly, the IR spectra confirm that PSI proteins remain in the film after electropolymerization of Py.

### Effect of PSI on PPy growth

**Fig. 3:**
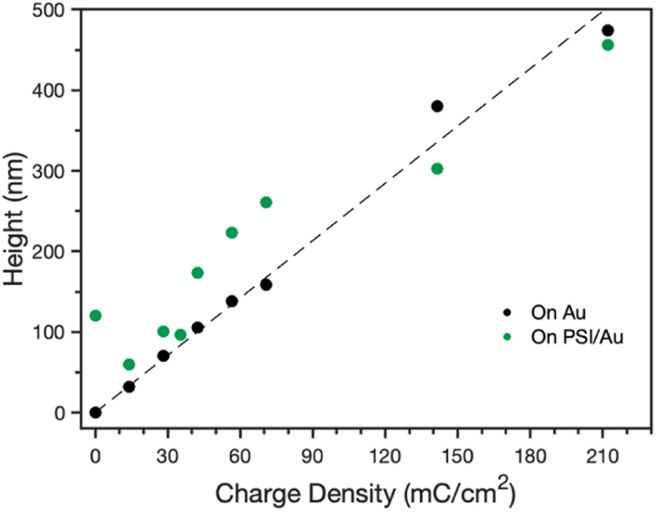
Effect of polymerization charge density on the base height for PPy grown on Au and PSI-modified Au electrodes. The base height is defined as the height above the Au substrate for contiguous film coverage. The dashed line is a best fit through the data for PPy grown onto Au.

After confirmation of the PSI-PPy composite film formation, profilometry was used to monitor the composite film thickness and topography as more PPy was incorporated into the film. Fig. S1‡ shows the profiles for Py electropolymerized on both bare gold (Au) and PSI-coated gold (PSI/Au), revealing that film thickness and roughness generally increase with additional polymerization charge. Fig. S1‡ also shows that the composite film is rougher than the pure PPy films are, which we attribute to the roughness of the drop casted PSI film and the likely heterogeneous initiation of PPy chains from less occluded regions of Au at the film/Au interface. From the film profiles, the base thicknesses of the film, or the height above the substrate for contiguous film coverage, was determined by averaging the lowest local minima across the scan (Fig. 3). For PPy grown on Au, the base thickness increases linearly with film charge up to 450 nm due to the homogeneous initiation across the electrode surface. For PPy grown on PSI/Au, the base thickness decreases at low levels of charge density passed, which we attribute to the initiation and early stages of PPy propagation disrupting thin regions of the PSI film. At intermediate levels of charge, the base thickness for PPy grown on PSI/Au increases sharply and then consistently with increasing charge as predominantly PPy domains begin to grow through the porosity of the rough PSI film. At high levels of charge, the base thickness exhibits a weak dependence on charge as the PPy grows past some PSI peaks and begins to grow laterally to spread along the surface and fill in voids. While the base thicknesses are similar for PPy on Au *versus* PSI/Au at the highest charge density of 210 mC cm^−2^, the total average thickness of the composite film is greater due to the presence of the protein in the film (Fig. S1d‡). The trends of base thickness and film morphology indicate that the PPy in the composite film is growing through and filling in void space within the PSI multilayer.

**Fig. 4:**
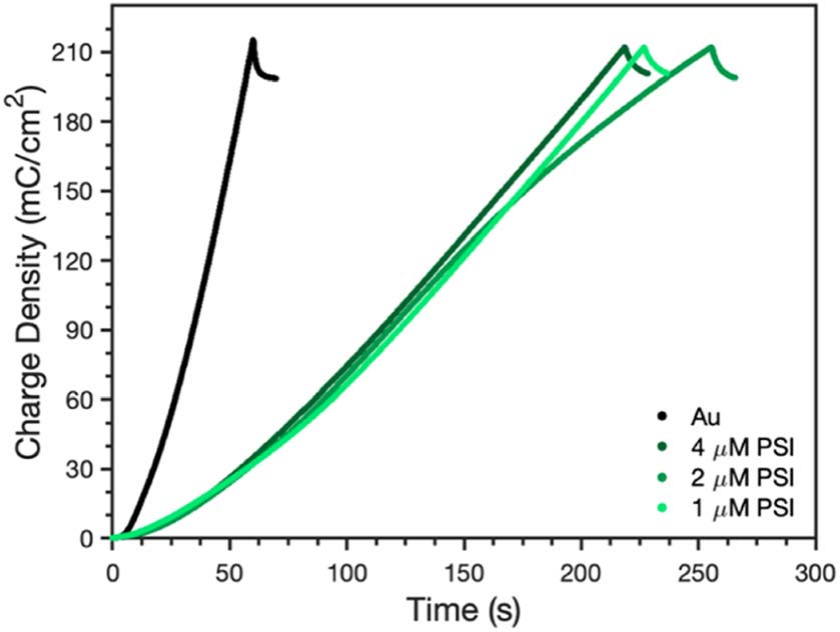
Time dependence of PPy growth on Au as quantified through charge density in the absence and presence of a PSI film. A 40 μL drop of PSI solution at the given concentrations was drop-cast for each film to vary the amount of PSI on the Au substrate. The applied voltage was 0.65 V *vs.* Ag/AgCl.

The rate of PPy growth was investigated by measuring the amount of charge passed at a set applied potential over time, which is directly related to the amount of polymer deposited.^34^ A multilayer of PSI proteins should reduce the PPy growth rate by hindering Py diffusion to the electrode and/or constraining film growth to certain regions of the electrode where the PSI film is thinner or highly porous because the protein itself is insulating.^35^Fig. 4 shows the time required to deposit 210 mC cm^−2^ of charge for polymerization of Py on gold, as well as gold coated with PSI films of varying thickness. The thickness was controlled by changing the concentration (1, 2, and 4 μM) of PSI in a 40 μL drop that was cast on an active circular surface area of 0.24 cm^2^. The concentration of PSI in the 40 μL drop is proportional to the thickness of the multilayer film on the surface since the deposition area is held constant.^36^ The presence of a PSI film has a dramatic effect on the polymerization rate. A charge density of 210 mC cm^−2^ was deposited after 60 s on gold but required 210–250 s (a ∼4-fold increase) once there was a film of PSI on the substrate. The total amount of PSI deposited had negligible effect on the growth rate, as PPy was able to grow through all PSI films similarly. This similarity in growth rates despite the PSI thickness indicates that PPy growth is limited to networks of voids in the PSI film, as similar PSI void fractions should exist near the electrode surface in all of these multilayer films. By limiting PPy growth through the voids of the PSI film, we expect that the composite film has PSI-rich and PPy-rich regions with some interfacial contact between the protein and polymer regions.

**Fig. 5:**
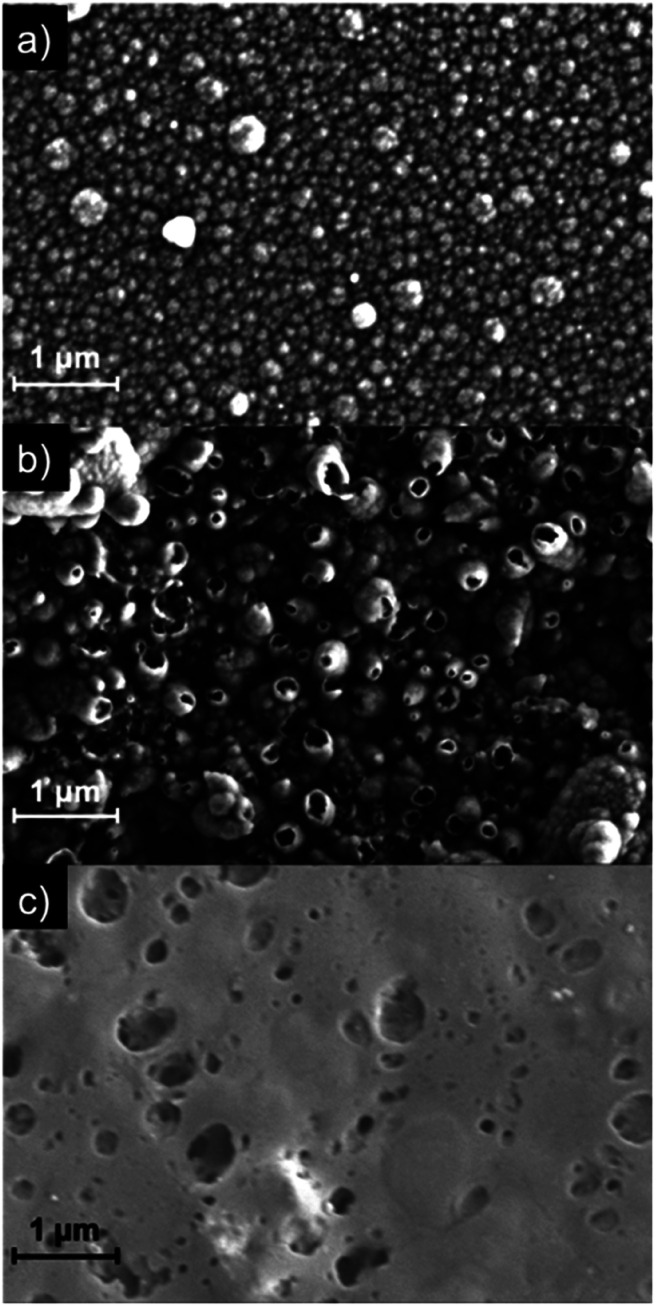
SEM images of PPy grown to a charge density of 210 mC cm^−2^ onto (a) Au and (b) PSI/Au. A drop-cast film of PSI (no Ppy) is shown in (c) as a comparison.

The SEM images in Fig. 5 compare the morphology of the composite PSI-PPy film with that of pure PPy at a common charge density of 210 mC cm^−2^, as well as a pure PSI film. The pure PPy film (Fig. 5a) shows a nodular cluster-like morphology with nodules ranging from 100–250 nm. This type of morphology is similar to that shown by us^33^ and others^37^ for electrochemically grown PPy. The image of the pure PSI control (Fig. 5c) shows a continuous but porous film in which the connected pores enable the diffusion of pyrrole monomers to access the near electrode regions. In contrast to both of these, the image of the composite film (Fig. 5b) shows many hollow tubule-like features with sizes ranging from 200–400 nm. These hollow structures are only seen when a PSI film is present and are consistent with PPy growth coalescing around dense PSI aggregates on the electrode surface. The protein aggregates thus appear to template the growth of the polymer on the substrate. Fig. 5 is visually consistent with the profilometry scans of Fig. S1,‡ which show a rougher surface for PPy grown on PSI/Au *versus* that on Au. Cross-sectional SEM images show that the composite film is much thicker than the PSI film (Fig. S2‡).

### Conductive nature of composite

**Fig. 6:**
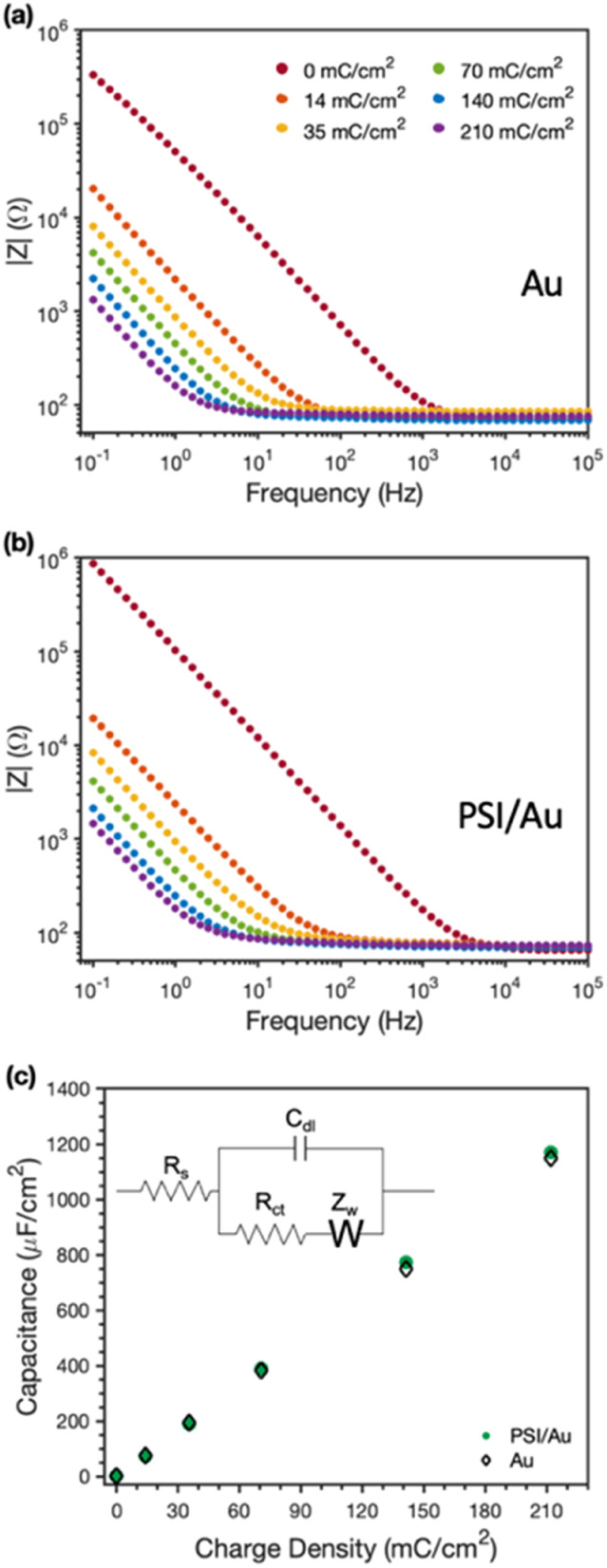
Electrochemical impedance spectra of (a) Au and (b) PSI/Au electrodes after electropolymerization of Py to the charge density indicated. Measurements were taken in an aqueous solution of 0.5 M KCl. (c) Effect of charge density of PPy deposited on the measured capacitance of the films on Au and PSI/Au. The equivalent circuit used to model the impedance spectra is shown as the inset in (c).

EIS was used to examine the conductive nature of the composite PSI-PPy films. Fig. 6 shows the EIS spectra with increasing amounts (charge deposited) of PPy grown at a constant applied potential on Au (Fig. 6a) and on PSI/Au (Fig. 6b). All impedance spectra show a solution resistance at high frequencies and a capacitance at intermediate to low frequencies. In both pure PPy and composite PSI-PPy films, the impedance decreases as more PPy is deposited, which is indicated by the lowering of the capacitive impedance at the low-to mid-range of frequencies in both plots. This lowering of the impedance is strikingly similar on both Au and PSI/Au and is due to the high capacitance of PPy, which has been ascribed by others to either the high interfacial area between conducting polymer fibrils and the internal electrolyte solution or charge transfer processes between the conducting polymer and the electrolyte (pseudocapacitance).^38,39^ The dramatic decrease in capacitive impedance (increase in capacitance) as more PPy is deposited means that the PPy is well connected to the gold electrode and functions as an extension of the electrode to complex ions from the electrolyte. Fig. 6b shows that a pristine PSI film tends to have a lower capacitance, and thus higher impedance, than those for films with conducting polymer present due to the insulating properties of the protein.^10,40,41^

The equivalent circuit model shown in the inset of Fig. 6c was used to fit the impedance spectra to quantify film capacitance, resistances due to solution and charge transfer, and the Warburg impedance (see Table S1‡). The most important of these for conducting films is the capacitance (Fig. 6c), which shows a nearly superimposable, linear relationship with charge density for PPy grown on both Au and PSI/Au electrodes. The similarity in capacitance values between the PPy and PPy-PSI films is consistent with the conducting PPy components in parallel with the insulating PSI components in the composite film. Also, since charge reflects the amount of polymer grown and capacitance is linear with charge for both the pure and composite films, we conclude that all the polymer grown is electrically connected and that the presence of PSI does not restrict the access of aqueous electrolyte to the internal surfaces of PPy fibrils.^10,39^ These observations further support the hypothesis that PPy is grown in voids between PSI clusters, and apparently with little, if any, mixing with the PSI protein within those clusters.

### Photoelectrochemical properties

The ability of these composite films to perform photoelectrochemistry was also investigated. Photocurrents were measured in an ascorbic acid, 2,6-dichlorophenolindophenol (AscH:DCPIP) mediator couple to investigate the photoelectrochemical properties of the protein-polymer films. In this mediator couple, ascorbic acid reduces DCPIP, which then functions as a fast donor to the P_700_ reaction center of PSI. As such, light triggers the rapid regeneration of DCPIP by PSI in the vicinity of the electrode; this DCPIP is then reduced rapidly at the electrode (Au or PPy) to yield a net cathodic photocurrent. Photocurrents were measured by illuminating the samples for 30 s and measuring the change in current before, during, and after illumination (see Fig. S2‡ for example PCA curves).

**Fig. 7:**
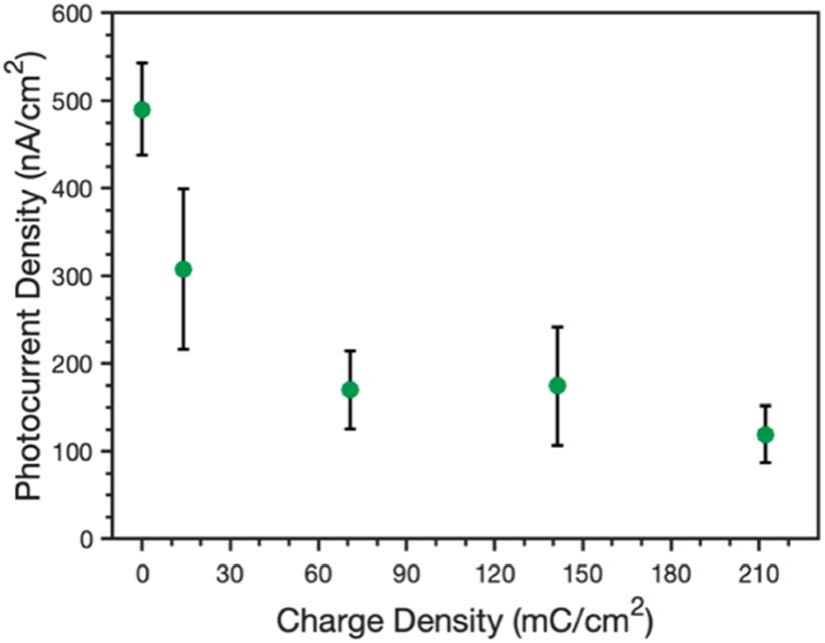
Effect of charge density of electrodeposited PPy on the peak photocurrent density of PSI/Au films. Measurements were made in 0.1 M KCl (aq) containing AscH/DCPIP (20 : 1 mM) as redox mediators. All films were illuminated for 30 s and the peak current typically occurred within the first 2 s.

Fig. 7 shows the peak photocurrents measured for an unmodified PSI film (no applied charge) as well as PSI films with increasing amounts of PPy grown, as directly related to the increasing charge density. Pure PPy shows no photoactivity with the AscH:DCPIP mediator on gold,^33^ but the addition of PSI results in cathodic photocurrent generation for all composite films. The photocurrent decreases sharply from 490 to 310 nA cm^−2^ after 14 mC cm^−2^ of PPy is grown and further drops to 170 nA cm^−2^ at and beyond 70 mC cm^−2^. The photocurrent is insensitive to greater amounts of PPy growth, just as the visual properties are similar for films at these same higher levels (Fig. 1b). There are likely contributing reasons for the drop in photocurrent. First, PPy does absorb some of the light that PSI requires for optimal photoactivity. Reflectance UV-vis spectra of the pure and composite films (Fig. S3‡) shows that PPy absorbs light across the visible region, including in the blue and red regions that are important for PSI photoelectrochemistry.^36^ Nonetheless, the fact that photocurrents are insensitive to higher loadings of Ppy suggests that this absorption of light is not the principal reason for the decrease in photocurrent. Second, while PPy provides a high interfacial area with the electrolyte, the polymer can hinder redox mediator transport to limit access to the active sites of PSI within the composite film. Thus, the rate that the mediator can remove holes from the P_700_ sites is reduced in the presence of the polymer, which can amplify charge recombination events^19^ within PSI clusters and decrease the cathodic current. Third, the large pseudo-capacitance of the film may slow redox-mediated charge transfer.

## Conclusions

PPy can be electropolymerized within the void spaces of a multilayer film of PSI to generate a conductive and photoactive composite film that has dense regions of protein and dense regions of polymer. During electropolymerization, the PPy grows around and between PSI clusters and fills in the voids of the protein film before continuing to grow beyond the PSI film. The presence of a PSI multilayer inhibits the polymerization rate of Py due to the insulating nature of protein clusters and their coverage of electrode area, but PPy is still able to initiate at less restricted gold surface regions and propagate through the voids in the protein film. The capacitance of both pure PPy and PPy/PSI films increases directly and identically with polymer charge, showing that the protein does not disrupt the interface between polymer fibrils and the internal electrolyte solution. The PSI-PPy films exhibit a photocurrent response that decreases as polymer is grown up to 70 mC cm^−2^ but levels off at higher polymer loadings. Nonetheless, a photocurrent response was achieved at all polymer loadings measured. This work shows that high loadings of PSI can be integrated with PPy at various loadings to produce a conductive, photoactive film.

The results from these straightforward composite films also show that phase-separated protein and polymer regions do not yield synergistic photoelectrochemical properties, in contrast to some other PSI-polymer composites in which better mixing of protein and polymer occur. In some other studies in which PSI has been combined with conducting polymers, most notably by entrapment of the protein with PAni,^9,32^ mixing of PSI with PEDOT:PSS,^31^ and layer-by-layer growth of PSI with PEDOT:PSS,^27^ the presence of the polymer has enhanced the measured photocurrent and the average electron turnover frequency of the PSI proteins. These enhancements have been attributed to interactions between the conducting polymer and individual protein redox centers that enable some direct electron transfer to occur. A similar argument has been made for the case of the photooxidized growth of PPy by PSI in solution in which conductivity of the resulting dry, packed powder increases in light.^33^ In contrast here, the presence of the PPy reduces the photocurrent of the composite film below that of the pure protein while still enabling a photoactive and conductive composite. A key difference here is that the PPy is grown through voids between large clusters in the PSI film, resulting in some peripheral PPy-PSI contact but limited interactions between the polymer and individual protein redox centers. This limited extent of interactions between polymer and proteins appears to be a key factor that precludes the synergistic effects observed in these other studies. Therefore, the future design of these PSI-polymer composite films and their methods of deposition should aim for well-mixed electronic and spatial interactions between protein and polymer.

## Experimental section

### PSI extraction

PSI was extracted from locally purchased spinach following a procedure described in a previous work.^42^ In short, the spinach was deveined, macerated, filtered, and then centrifuged at 8000*g* to isolate the thylakoid membranes. The supernatant was then mixed with a surfactant (TritonX-100) to lyse the membranes before a second centrifugation at 20 000*g*. A hydroxyapatite column was used to isolate the PSI. The protein was dialyzed in deionized water at a 1000 : 1 volume ratio for 12 h in a 10 000 MWCO membrane to remove surfactant and excess salt. A visible absorption spectrum^36^ and SDS-PAGE^33^ analysis were performed previously on PSI prepared in this manner.

### PPy electropolymerization around PSI multilayers

PSI multilayer films in this work were all deposited using a vacuum-assisted drop casting method. To prepare the films, an electrochemical mask was placed on the desired substrate. Before pressing onto the substrate, a central hole of 0.24 cm^2^ was cut from the mask to yield consistent active areas for the resulting films. Dialyzed PSI solution was deposited onto the substrate in a 40 μL drop and placed under vacuum to increase the drying rate of the film. A multilayer film of PSI remained after evaporation of all the water. Once dry, the films were rinsed in water to remove any excess salt and then dried under vacuum again. The PSI multilayer film was then placed in an electrochemical cell with a 0.5 M pyrrole solution with 1.0 M NaClO_4_ as a doping electrolyte. To perform the polymerization, the gold electrode was biased to 0.65 V *vs.* Ag/AgCl to initiate electropolymerization of pyrrole on the gold surface, and a set amount of charge was applied to ensure consistent amounts of polymer were formed.

### Photochronoamperometry (PCA)

The photoactivity of PSI protein films was measured through PCA. PCA is a specialized form of chronoamperometry, wherein a working electrode is held at a measured open-circuit potential, and the current passed through the electrode is measured over time. For PCA experiments, working electrodes were held at the OCP measured in dark conditions, and the current response of the electrode was measured upon illumination of the electrode with white light for 30 s using a Leica KL 2500 LCD cold light source (140 mW cm^−2^). All PCA experiments were performed on a CH Instruments 660a electrochemical workstation. The photocurrents were measured in 0.1 M KCl (aq) with a 20 mM ascorbic acid, 1 mM 2,6-dichlorophenolindophenol (AscH:DCPIP) mediator couple.

### Electrochemical impedance spectroscopy (EIS)

EIS was used to measure electrochemical properties for Ppy films with and without PSI multilayers. EIS measurements were measured in 0.5 M KCl solution at the open circuit potential with a 10 mV sinusoidal variation from frequencies of 0.1 to 100 000 Hz on a Gamry Reference 600 potentiostat. The working electrode was the film-coated gold surface, the counter electrode was an uncoated gold electrode, and the reference electrode was Ag/AgCl (sat'd KCl).

### ATR FTIR

Attenuated total reflectance Fourier transform infrared spectroscopy (ATR-FTIR) was performed to determine the chemical compositions of the protein, polymer, and composite films using a Thermo Nicolet 6700 FT-IR spectrometer equipped with a liquid-nitrogen cooled mercury–cadmium–telluride (MCT) detector and Smart iTR™ ATR attachment with a diamond crystal plate. The spectra were collected in the region of 4000–700 cm^−1^ over 256 scans at 4 cm^−1^ resolution and processed using OMNIC™ software.

### Profilometry

Profilometry was performed using a Veeco DEKTAK 150 stylus profilometer to probe the 2D topography of films on gold substrates. A light source was aimed at the top of the cantilever, and the changes in light deflections as the tip scans across the surface were collected to measure the height of a film at a resolution <100 nm. The scans were recorded over 4 mm using a stylus with a 12.5 μm radius, applying 2.0 mg force, and employing a hills-and-valleys detection mode.

Base thicknesses of the PPy and composite films were determined through a series of functions run on the profilometry data. First, every local minimum of the data set was determined. Then, a base thickness was estimated by computing the average of the lowest local minima in different regions across the profile.

### SEM

Scanning electron microscopy (SEM) was conducted using a Zeiss Merlin equipped with a Gemini II Column with an accelerating voltage of 2.00 kV. Non-conductive samples were gold sputtered for 20 s in an argon environment prior to imaging.

### Reflectance UV-vis

Ultraviolet visible (UV-vis) spectroscopy with a scan range of 350–800 nm was performed using a Varian Cary 5000 UV-Vis-NIR spectrophotometer operating in dual beam mode with a specular reflectance accessory. The scan rate was set to 300 nm min^−1^, and a bare gold substrate was used as the baseline reference sample.

## Author contributions

J. M. Passantino: investigation, methodology, writing-original draft. B. A. Christiansen: investigation, writing – original draft. M. A. Nabhan: investigation, writing-review and editing. Z. J. Parkerson: investigation, writing – review and editing. T. D. Oddo: investigation, writing-review and editing. D. E. Cliffel: conceptualization, writing-review and editing. G. K. Jennings: conceptualization, supervision, writing-review and editing.

## Conflicts of interest

There are no conflicts to declare.

## Supplementary Material

NA-005-D3NA00354J-s001
