# Supplementary material for: Photoactive and conductive biohybrid films by polymerization of pyrrole through voids in photosystem I multilayer films
Source: Nanoscale Adv. 2023 Aug 26;5(19):5301–8. doi: 10.1039/d3na00354j (PMC10521210; doi:10.1039/d3na00354j)
Supplement: NA-005-D3NA00354J-s001 [file NA-005-D3NA00354J-s001.pdf]

Electronic Supplementary Material (ESI) for Nanoscale Advances.
This journal is © The Royal Society of Chemistry 2023

Electronic Supplementary Information

Photoactive and Conductive Biohybrid Films by Polymerization of Pyrrole through Voids in PSI Multilayer Films

Joshua M. Passantino,[1] Blake A. Christiansen,[1] Marc A. Nabhan,[1] Zane J. Parkerson,[1] Tyler D. Oddo,[1] David E. Cliffel,[2] and G. Kane Jennings[1,*]

[1] Department of Chemical and Biomolecular Engineering, Vanderbilt University, Nashville, TN 37235-1604

[2] Department of Chemistry, Vanderbilt University, Nashville, TN 37235-1822

* Author to whom correspondence should be addressed.

Table of Contents

Figure S1. Surface profiles for Ppy grown on Au and PSI/Au...

Figure S2. Cross-sectional SEM images for PSI and PSI-PPy films…

Table S1. Electrochemical parameters of Ppy films grown to the charge densities indicated onto Au and PSI/Au…

Figure S3. Sample PCA of a PSI film and composite PSI-PPy films…

Figure S4. Reflectance UV-Vis spectra of PSI, PPy, and a composite PSI-PPy film on Au…

Profilometry. Figure S1shows surface profiles for PPy grown on Au and PSI/Au to different levels of charge. On Au, PPy exhibits a clear base thickness that increases with higher levels of polymerization (charge). The polymer films are rougher at higher levels of polymerization. For PPy grown on PSI/Au, the initial PSI film is very rough, and as PPy polymerization proceeds to higher levels of charge, the base thickness increases while the high level of roughness remains. At the highest charge, comparing profiles for PPy on Au versus that on PSI/Au, the roughness is apparently a combination of that due to PSI and PPy.

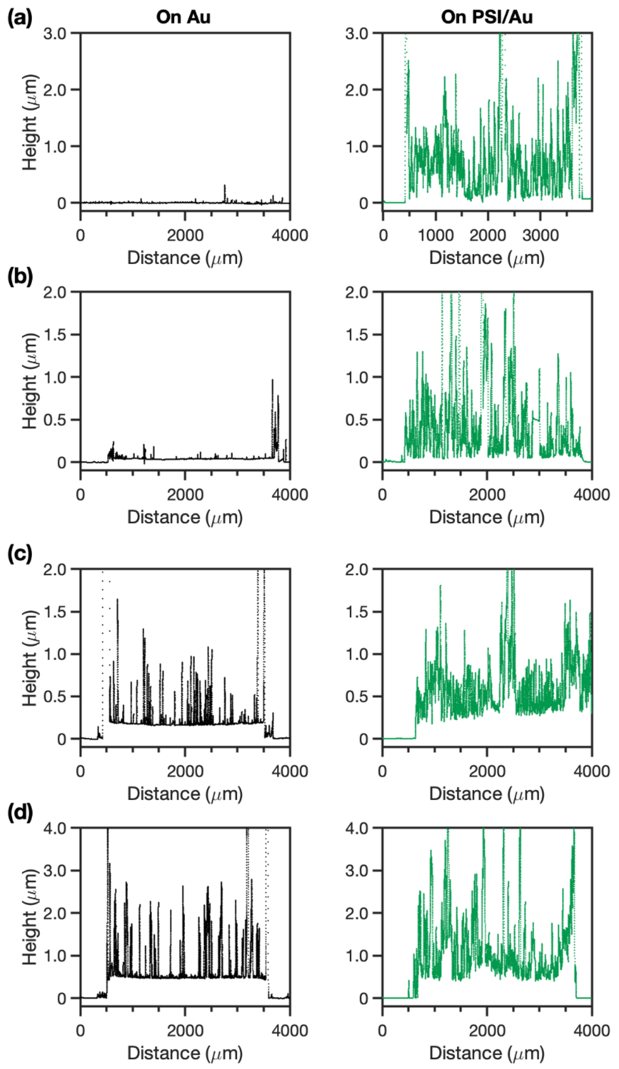

***Figure S1**. Surface profiles for Ppy grown on Au and PSI/Au at (a) 0 mC/cm^2, (b) 14 mC/cm^2, (c) 70 mC/cm^2, and (d) 210 mC/cm^2.*

Cross-Sectional Scanning Electron Microscopy (SEM). To provide additional information on the morphology of the films, Figure S2 shows cross-sectional SEM images of a pure PSI film and a PSI-PPy composite film where the polymer was deposited at 70 mC/cm^2 charge density. Both films are rough, but the composite film is significantly thicker due to the loading of the PPy. We

were unable to distinguish PPy or PSI regions of the film through either these images or energy-dispersive spectroscopy.

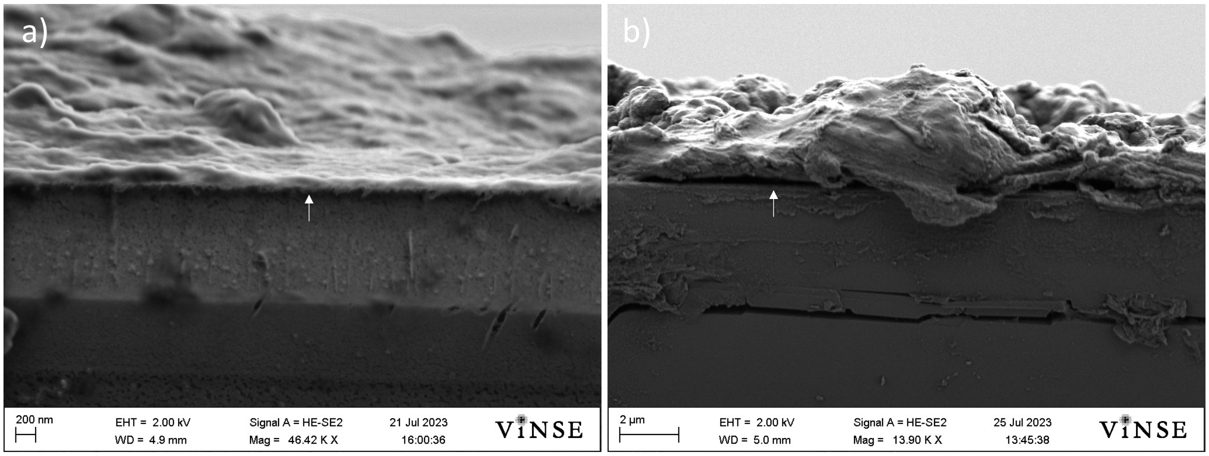

***Figure S2**. Cross-sectional SEM images of (a) a PSI film, and (b) a composite PSI-PPy film at 70 mC/cm^2 charge density. White arrows indicate the approximate floor of the film.*

Fitted Parameters from Electrochemical Impedance Spectroscopy (EIS). The electrochemical impedance spectra in Figures 6a and 6b of the main text were fit with the equivalent circuit in the inset of Figure 6c to determine electrochemical parameters. Table S1 shows double layer capacitance (C_{dl}), charge transfer resistance (R_{ct}), and the Warburg coefficient (σ) for the spectra in Figures 6a and 6b.

***Table S1.** Electrochemical parameters of Ppy films grown to the charge densities indicated onto Au and PSI/Au. Parameters were determined by fitting the impedance spectra in Figure 6 to the equivalent circuit in Figure 6c .*

	Au						PSI/Au					
**Charge (mC/cm^2)**	**0**	**14**	**35**	**70**	**140**	**210**	**0**	**14**	**35**	**70**	**140**	**210**
C_{dl} (µF/cm^2)	3.0	75	200	380	750	1200	1.4	73	190	390	770	1200
R_{ct} (kΩ)	730	180	96	86	70	89	1900	130	81	66	190	210
$\sigma^{\dagger}$ (Ω s$^{-1/2}$)	1700	200	83	50	33	32	3200	530	210	110	53	47

† Warburg coefficient

Example Photochronoamperometry (PCA) Results. Figure S3 provides examples of PCA results for a film of PSI as well as films in which PPy is grown to different charges through voids in the PSI film. The film was initially in the dark, and light was irradiated on the sample from 20 to 50 s. The measurements were made in a 0.1 M KCl aqueous solution of 20 mM ascorbic acid (AscH) and 1 mM 2,6-dichlorophenolindophenol (DCPIP) with an incident white light intensity of 140 mW/cm^2. Figure S3 shows that the initial spike in cathodic current is reduced if PPy is deposited into the film. We attribute this reduction in peak current to the effect of PPy in limiting mediator access to the PSI active sites and in reducing the light that PSI is able to absorb. Figure S3 also shows that the baseline current after illumination ends is anodic, and generally more anodic if Ppy is present, suggesting that the larger electrode surface area (gold plus PPy) versus that of gold alone effectively alters the balance of reduced and oxidized species during illumination.

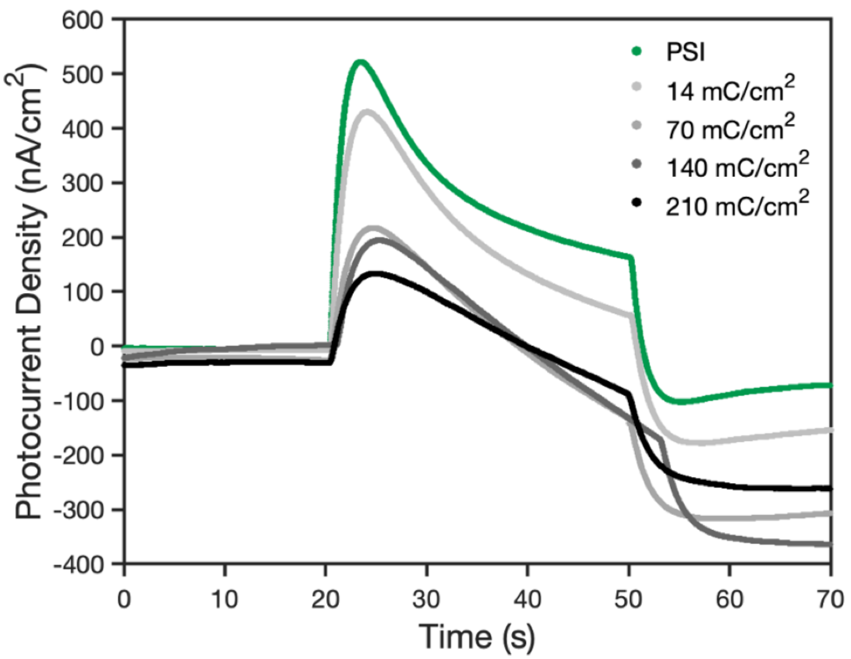

Figure S3. *Sample PCA of a PSI film and composite PSI-PPy films. Measurements were made in AscH/DCPIP mediator solution. Samples were illuminated starting at 20 s and ending at 50 s.*

Reflectance UV-Vis Spectroscopy. Figure S4 shows reflectance uv-vis spectra for gold substrates containing a PSI film and an electrodeposited PPy film at 270 mC/cm^2 of charge, as well as a PSI-coated gold substate with an electrodeposited PPy film at the same charge density. PSI shows absorbance peaks around 430 nm and 670 nm due to α-chlorophylls in the protein. PPy alone absorbs broadly across most of the visible region, thus suggesting that the polymer absorbs light that could be used by the protein when the two components are combined in the composite. For the PSI-PPy composite, we observe elevated light absorbance from 480 – 730 nm that could be attributed to component absorbances from both PPy and PSI.

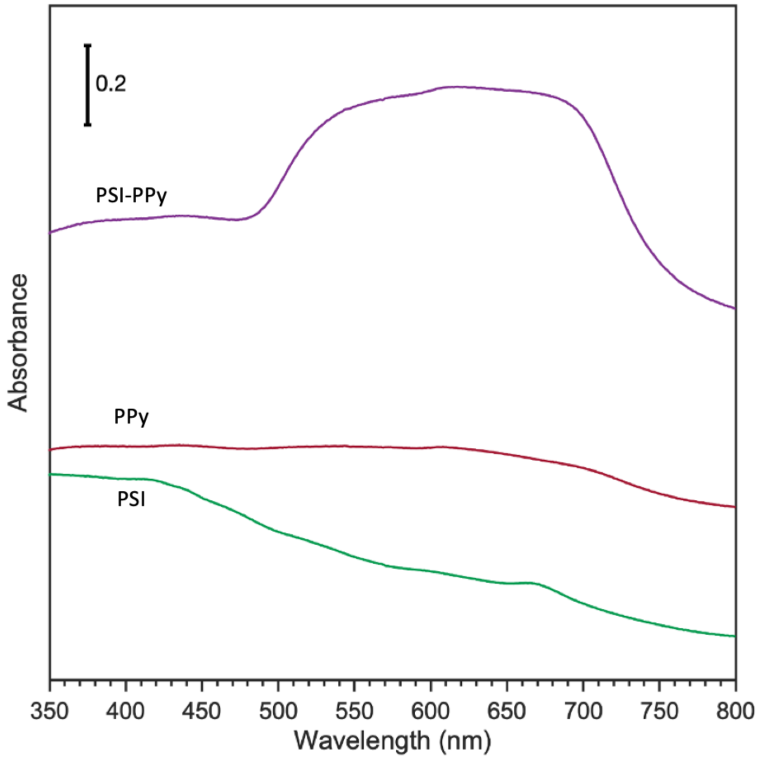

Figure S4. *Reflectance UV-Vis spectra of PSI, PPy, and a composite PSI-PPy film on Au. 210 mC/cm^2 of polymer was deposited for both the PPy and composite films.*
